# HFAC Dose Repetition and Accumulation Leads to Progressively Longer Block Carryover Effect in Rat Sciatic Nerve

**DOI:** 10.3389/fnins.2022.852166

**Published:** 2022-05-27

**Authors:** Adrien Rapeaux, Timothy G. Constandinou

**Affiliations:** ^1^Next Generation Neural Interfaces Lab, Centre for Bioinspired Technology, Department of Electrical and Electronic Engineering, Imperial College London, London, United Kingdom; ^2^Care Research and Technology Centre, UK Dementia Research Institute, London, United Kingdom

**Keywords:** stimulation, nerve, alternating current, HFAC, block, *ex-vivo*, carryover, high frequency

## Abstract

This paper describes high-frequency nerve block experiments carried out on rat sciatic nerves to measure the speed of recovery of A fibres from block carryover. Block carryover is the process by which nerve excitability remains suppressed temporarily after High Frequency Alternative (HFAC) block is turned off following its application. In this series of experiments 5 rat sciatic nerves were extracted and prepared for *ex-vivo* stimulation and recording in a specially designed perfusion chamber. For each nerve repeated HFAC block and concurrent stimulation trials were carried out to observe block carryover after signal shutoff. The nerve was allowed to recover fully between each trial. Time to recovery from block was measured by monitoring for when relative nerve activity returned to within 90% of baseline levels measured at the start of each trial. HFAC block carryover duration was found to be dependent on accumulated dose by statistical test for two different HFAC durations. The carryover property of HFAC block on A fibres could enable selective stimulation of autonomic nerve fibres such as C fibres for the duration of carryover. Block carryover is particularly relevant to potential chronic clinical applications of block as it reduces power requirements for stimulation to provide the blocking effect. This work characterizes this process toward the creation of a model describing its behavior.

## 1. Introduction

The holy grail of neural interfaces is often thought of as “one to one” interfacing with nerve cells such that perfect control of the interface between the nervous system and a therapeutic or communication device can be achieved. It is evident that the field is still a long way from this goal; current stimulation-based therapies all have side-effects tied to the non-selective nature of the interface used to deliver the therapy (Bonaz et al., 2017; Peh et al., 2018). For example, neural prostheses that attempt to restore the sense of touch with electrical stimulation have to selectively activate specific nerve fibre populations within complex nerve trunks (Graczyk et al., 2016), and nerve stimulators for bladder dysfunction have to activate nerve fibres leading to bladder wall contraction, while minimizing activation of other fibres leading to the external urethral sphincter (McGee et al., 2015). In autonomic neuromodulation it is known that vagus nerve stimulation has side-effects deriving from lack of selectivity (Guiraud et al., 2016). Target and off-target fibres are often located in the same nerve trunk, meaning that improving selectivity by changing the surgical location of stimulation electrodes is often not possible. This has led to significant effort to develop means to selectively stimulate specific fibres in whole peripheral nerve.

Attempts to improve electrode selectivity to reduce stimulation therapy side-effects include development of more invasive electrodes that place miniaturized contacts closer to the neuron axons, within the perineurium or endoneurium in peripheral nerve (Rijnbeek et al., 2018). This often leads to increased foreign body reactions and lower implant lifetime compared to when using cuff electrodes, the peripheral nerve interface type with the least long-term complications. In order to avoid using more invasive interfaces, another means to improve selectivity is to leverage different neuromodulation techniques and combine them. Carefully designed stimulation waveforms can yield selective effects such as unidirectional CAP propagation, for example (Grill and Mortimer, 1995), however these haven't entered clinical practice. A promising new tool for neuromodulation is high frequency block, which enables stimulation to reversibly inhibit nerves and suppress their activity rather than exciting them (Bhadra et al., 2018). Using HFAC block and conventional stimulation together could improve the selectivity of low-invasiveness electrode interfaces such as cuff electrodes, for example in bladder control (McGee et al., 2015; Rapeaux et al., 2015) however the processes and mechanisms involved with HFAC block are still not completely understood.

One such mechanism is so-called “block carryover”, as reported in Waataja et al. (2011); Liu et al. (2013); Bhadra et al. (2017); Yang et al. (2017), and Pelot and Grill (2020), describing a process by which nerve excitability remains suppressed after application of HFAC block. This effect is not captured by computational simulations of nerve cells being stimulated by high-frequency signals (Ackermann et al., 2011; Liu et al., 2013; Pelot et al., 2017; Perra et al., 2018), requiring characterization before HFAC stimulation can be used in a clinical context. Furthermore, previous experimental work with HFAC has not always observed carryover block (Kilgore and Bhadra, 2004), indicating that the conditions in which carryover block occurs may be unclear and require clarification. As carryover has been observed for A fibres in peripheral nerve, this could provide an opportunity to selectively block A fibres and stimulate autonomic nervous system fibres such as C fibres in the same nerve trunk. This is in contrast with nerve recruitment order from large to small diameters when using conventional stimulation, wherein activation of C fibres requires activation of A fibres within the same trunk. In stimulation therapies targeting nerve trunks with mixed fibre compositions such as the vagus nerve, and where stimulation of C fibres are required for efficacy (Chang et al., 2020), concurrent A fibre activation results in painful muscle contractions as a side-effect. C-fibre selective stimulation, even if only during limited time windows, would make these therapies practical and tolerable in a clinical scenario. The work of Waataja et al. (2011) supports the hypothesis that C fibres recover more quickly from block than larger fibre types, however it remains to be demonstrated whether those observations can be reproduced in other nerves of the rat, and furthermore in humans.

The duration of block carryover varies substantially depending on the report, with Pelot and Grill (2020) citing tens of seconds, and Bhadra et al. (2018) citing hours in some cases. This seems to be dependent on the amount of HFAC block that was applied to the nerve before cutoff (Waataja et al., 2011). In all reports nerve activity returns to baseline, suggesting that this process is not the result of nerve damage. Previous work has so far predominantly used experimental setups with one nerve either *in-vivo* or *ex-vivo*, with at one extremity a pair of stimulation electrodes, at the other a pair of recording electrodes (or in the *in-vivo* case, sometimes a load cell attached to a muscle) to measure nerve excitability, and between them a bipole or tripole used to deliver HFAC stimulation. Previous works are summarized in terms of animal used and reported carryover duration in Table 1 below. Some previous works do not record C fibre activity, and therefore only provide information on recovery times of A fibres from block.

**Table 1:** Previous works reporting carryover duration and animal model.

**References**	**Animal**	**Nerve target**	**Carryover duration**
Waataja et al. (2011)	Rat (*ex-vivo*)	Vagus	4–10 min
Liu et al. (2013)	Bullfrog (*ex-vivo*)	Sciatic	Seconds to minutes
Yang et al. (2017)	Frog (*ex-vivo*)	Sciatic	50 to 450 s
Bhadra et al. (2017)	Rat (*in-vivo*)	Sciatic	minutes to hours
Rapeaux et al. (2018)	Rat (*in-vivo*)	Sciatic	20–430 ms
Pelot and Grill (2020)	Rat (*in-vivo*)	Vagus	0–100 s

The variability of carryover duration reported in different works, sometimes with the same nerve and in the same animal warrants further investigation. This work aims to determine whether carryover can be reproduced in the sciatic nerve of the Sprague-Dawley rat, a useful model as the sciatic nerve contains both A and C fibres and selective stimulation can be tested.

The remainder of this paper is organized as follows: Section 2 describes the methods used to explore HFAC carryover, including an overview of the *ex-vivo* experimental platform used for the experiments, and the specific stimulation protocol used to obtain the observations. Section 3 presents the experimental results. Section 4 discusses the results, potential limitations of the protocol, and several observations that could not be readily explained at the time these experiments were carried out. Finally Section 5 concludes on observed and previously undescribed behavior for HFAC block carryover that will inform future work and pave the way toward clinical applications of HFAC block that leverage this process.

## 2. Methods

### 2.1. Approach

While carryover itself has been previously reported, it is essential to be able to predict its duration for clinical application of this process. The first step is to determine how consistent the effect is, particularly when block is applied repeatedly. Therefore experimental design focused on measuring whether carryover duration was affected by repeated applications of the same HFAC block stimulation protocol, and verifying whether HFAC dosage would change carryover duration as was previously reported.

### 2.2. Experimental Neurophysiology Setup

**Figure 1:**
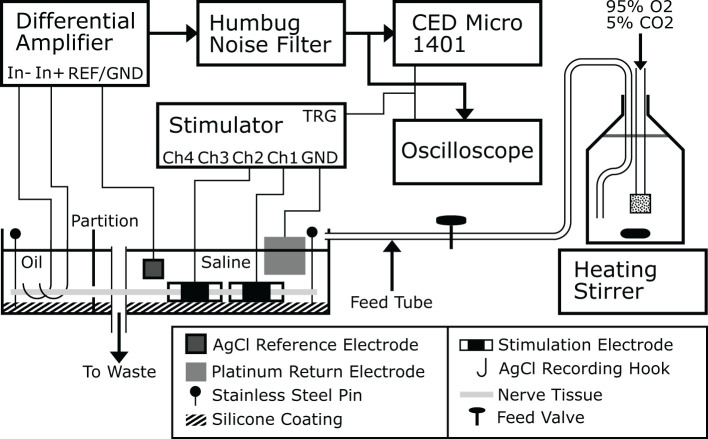
A drawing of the *ex-vivo* experimental platform used to carry out the experiments. Buffer is prepared and stored in a large bottle that is continually stirred, aerated and heated. Buffer flows from the bottle to the nerve bath continuously and drains to waste, or alternatively directly back to the bottle itself. This figure was previously published in Rapeaux and Constandinou (2020).

Experiments were carried out *ex-vivo* in a carefully controlled environment for the nerve. Nerve tissue homeostasis was maintained by bathing the nerve in modified Krebs-Henseleit Buffer (mKHB), with its composition specified in Table 2. The bath was continually perfused with fresh buffer pre-heated to 37 degrees Celsius, and perfusion is carried out by gravity feed using a siphon, as shown on Figure 1. Buffer in the primary container was continually aerated with a mixture of 95% dioxygen and 5% carbon dioxide, also called carbogen, ensuring adequate oxygenation of the tissue in the bath while buffering pH using the mKHB's carbonate-based pH buffering mechanism. Carbogen is provided by a canister located next to the setup.

**Table 2:** Ion and sugar composition of mKHB in [mmol.L^−1^].

**Ion**	**mKHB**
Na^+^	138	
Cl^−^	122.8	
K^+^	6	
Ca^2+^	2.5	
${\text{PO}}_{4}^{2-}$	1.2	
Mg^2+^	1.2	
${\text{SO}}_{4}^{2-}$	1.2	
${\text{CO}}_{3}^{2-}$	25	
Glucose	5.55	

During a typical experiment, recording is carried out using a bipolar pair of silver silver-chloride hook electrodes in contact with the nerve tissue in the oil. The signal is first amplified using an SRS-560 low-noise differential amplifier (Stanford Research Systems, USA) with its gain set to 100 and its second order bandpass filter set to between 10 and 1,000 Hz. A reference silver-silver chloride electrode in the bath is connected to the amplifier's isolated ground contact. The preamplifier signal is passed through a Humbug line-noise eliminator (Digitimer, UK) to remove 50 Hz line noise. The signal can be viewed by an oscilloscope triggered by the stimulator and is recorded to disk by a Micro-1401 multichannel ADC (CED, UK). Further filtering of the waveform was applied during post-processing within the Spike2 software package as needed to remove stimulation artifacts. The stimulator used with the setup is the custom-made 4-channel block capable stimulator previously described (Rapeaux and Constandinou, 2020), however any nerve stimulator with a TTL trigger input or output can be used, such as a DS3 (Digitimer) (Patel et al., 2017). For HFAC stimulation a current waveform generator such as a Keithley 6221 (Keithley Instruments) and DC-canceling output filter can be used (Pelot and Grill, 2020). Each stimulator channel connects to one contact of a stimulation electrode, while a large platinum sheet is used as a common return in the bath with no influence on nerve activation.

Stainless steel insect pins are used to secure the nerve to an inert silicone coating on the bottom of the bath, ensuring consistent contact of the nerve with the suspended recording electrodes. This however requires the oil and aqueous phases to be at the same level to allow the nerve to rest flat. For this purpose the nerve is threaded through a partition in the bath separating the oil and buffer compartments, and silicone grease is applied using a large bore syringe around the nerve on both sides of the partition to seal both compartments and prevent leakage of liquid from one into the other. The placement of multiple stimulation electrodes allows concurrent stimulation and block to be applied; generally the stimulation electrode is placed at the extremity of the nerve and the blocking electrode between the stimulating electrode and recording hooks. In this way the effect of block on evoked nerve action potentials can be accurately measured. The orientation of the nerve in the bath is determined depending on whether efferent or afferent nerve conduction is to be studied. To replicate *in-vivo* studies as closely as possible, stimulation electrodes are wrapped around the nerve in the saline compartment of the bath.

### 2.3. Custom Conductive Elastomer Electrode for HFAC Block

**Figure 2:**
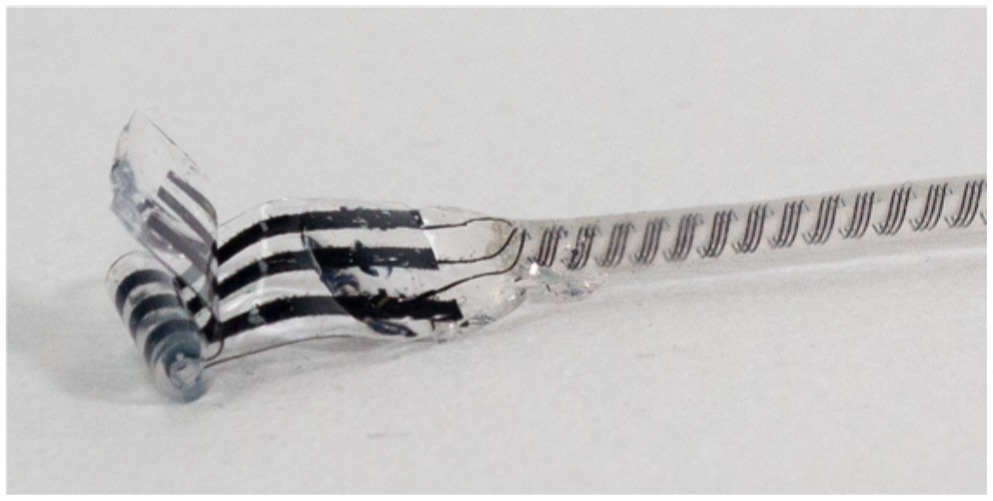
Elastomer electrode used in *ex-vivo* block and stimulation experiments at Imperial College. The conductive elastomer is black and encased in flexible silicone, with a silastic tube to shape the material into a cylinder for use as a cuff electrode. Material and electrode manufacturing details are provided by Cuttaz et al. (2021). Courtesy of Estelle Cuttaz, Rylie Green research group, Imperial College.

As HFAC block uses much more intense stimulation currents than conventional stimulation, it is important to choose an electrode that will not damage the nerve by producing reactive oxygen species, toxic metal salts, or change local pH when large currents are sourced or sunk through it. The maximum currents that can be handled by conventional platinum electrodes without reaching water-splitting polarization levels is known to be limited (Ghazavi and Cogan, 2018). In this work, we used a custom electrode made from conductive elastomer (Cuttaz et al., 2019) to deliver HFAC block. These electrodes have individual contact areas of 3.14 mm^2^, and the maximum currents used were 6 mA, therefore the maximum current densities were 191.1 mA/cm^2^ during HFAC block. This electrode is made from conductive traces of polymer sandwiched by silastic backing. A slit silastic tube is fitted over the composite in order to obtain a cuff electrode shape. Windows are cut out of the inner silastic layer to expose the traces of conductive polymer below, which makes contact with the nerve tissue. A photograph of the electrode is shown in Figure 2.

### 2.4. Tissue Preparation Protocol

All animal care and procedures were performed under appropriate licences issued by the UK Home office under the Animals (Scientific Procedures) Act (1986) and were approved by the Animal Welfare and Ethical Review Board of Imperial College London. In the following experiments, the following protocol was used for preparation of the rat sciatic nerve for recording and stimulation:

Briefly, Sprague-Dawley rats were anesthetized using isoflurane. Once deep anesthesia was obtained and verified by an absence of reflex to noxious toe pinch stimuli, animals were culled by cervical dislocation. Animals were placed dorsal side up on a dissection table. First the calcaneal tendon is cut to gain access to the space between the skin and leg muscles. Blunt dissection scissors are used to cut through the skin of the leg from the ankle upwards to the spine following the tibia and femur of the outstretched leg. The sciatic nerve was carefully exposed by cutting through the muscle planes, and the resulting cavity immediately moisturized using mKHB cooled on ice. The *gastrocnemius medialis* muscle is split to expose the complete tibial branch of the sciatic nerve. The nerve is then carefully removed from the leg, starting with the distal end of the tibial branch at the ankle, and upwards as close to the spine as timely dissection allows. This should yield a section of nerve approximately 5 cm long in about 5–10 min of dissection per leg, where speed is important for preservation of tissue integrity. All branches of the sciatic nerve except the tibial branch are cut and the nerve placed in cold mKHB for transport. Under a dissection microscope the nerve is placed in a silicone-coated petri dish and both ends are ligated using 6-0 sutures. The nerve is carefully cleaned of any residual fascia, blood vessels, muscle tissue, and auxiliary nerve branches are trimmed close to the trunk to prevent variation in contact quality with cuffs placed on the main nerve trunk. The prepared nerve is then placed in the nerve bath with warmed oxygenated and pH controlled mKHB buffer in one compartment and mineral oil in the other to begin stimulation and recording. Stimulation electrodes are carefully wrapped around the nerve to prevent kinking or pinching. Nerves are expected to remain viable for experiments for up to 7–8 h after dissection when placed in adequate homeostasis (Bailey and Ong, 1978; McAlexander et al., 2014).

### 2.5. Stimulation Protocol

A set of experiments was designed to investigate carryover for selective stimulation. The primary scientific question was whether carryover could be induced in A fibres but not C fibres, or whether C fibres would recover from carryover more quickly as reported by Waataja et al. (2011) but this time in the rat sciatic nerve.

Initial work suggested that HFAC block delivered with a 10 kHz 6 mA square current waveform was effective for blocking A fibres reversibly. In total 5 experiments were carried out on 5 different nerves to characterize carryover in the rat sciatic nerve. Block was delivered using the conductive elastomer electrode previously described, and stimulation was delivered using a conventional cuff electrode with platinum contacts.

**Figure 3:**
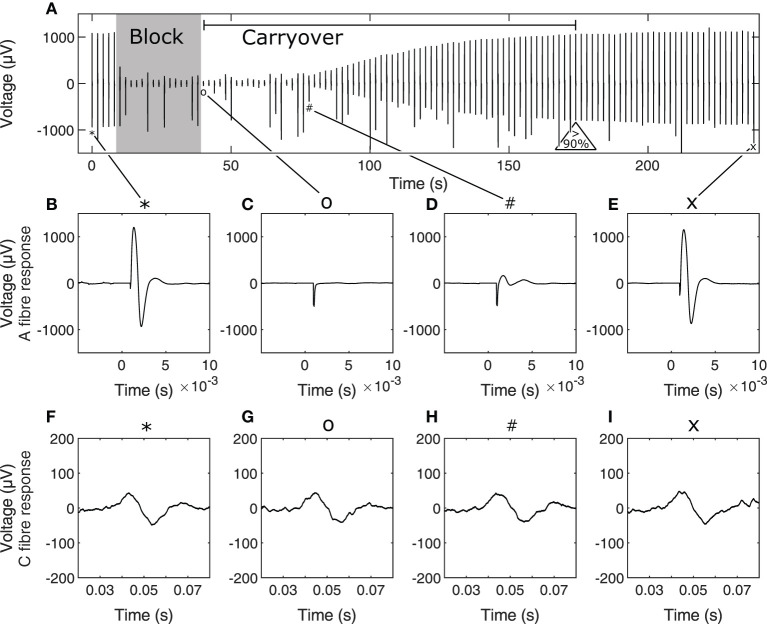
Example stimulation and block carryover measurement trial timeline **(A)**, showing peak-peak values of the nerve response to each stimulus during the trial. The 30-s block period and carryover period, determined as the period between end of block and recovery to 90% of baseline A fibre activity are annotated on **(A)**. Initial baseline stimulation for A fibres is shown in **(B)** and C fibres in **(F)** at stimulation event 1, A and C fibre response immediately after end of block in **(C,G)**, respectively, at stimulation event 21, A and C fibre response at the start of A fibre recovery from carryover in **(D,H)**, respectively, at stimulation event 40, and A and C fibre response toward the end of carryover recovery in **(E,I)**, respectively, at stimulation event 120. Corresponding stimulation events in the timeline are tied to their corresponding A and C fibre response in zoomed captures using symbols on the figure. Occasional jumps in signal peak-to-peak values are due to spurious noise during the recording. Note that time scales for A and C fibre responses are different. In **(B–I)**, time scales are relative to the time of stimulation for the corresponding nerve response.

For each trial within an experiment, stimulation was first delivered 5 times to establish a baseline for nerve excitability. Stimulation pulse width and amplitude were adjusted to recruit both A and C fibres. Stimulation waveforms were biphasic symmetric starting with a cathodic phase of 300 μs duration and 5 mA amplitude, followed by 20 μs of interphase before the anodic phase. Block was delivered for 30 or 60 s at 6 mA amplitude and 10 kHz depending on the trial to determine whether carryover duration depended on HFAC block dose independently of block signal amplitude and frequency. This was sufficient to reach supramaximal levels of nerve recruitment as initial tests showed further increases in stimulation amplitude did not result in increases in CAP peak-to-peak values. Block and carryover trials were repeated 3 or 4 times in order to determine whether carryover duration changed depending on accumulated dose of HFAC block. Subsequent sets of trials were delivered using other contacts of the three-contact conductive elastomer electrode to determine whether carryover duration was affected by contact location. Inter-contact distance was 2 mm. Recovery was considered achieved when A fibre activity recovered to at least 90% of baseline. Time to recovery is the time between block cutoff and reaching this threshold. Not all block trials led to measurable carryover due to limitations in how often the nerve was stimulated during the trials, which was once every 2 s—in some cases the nerve recovered to more than 90% of baseline activity from one stimulus to the next at the start of recovery. In total 26 out of 48 trials resulted in measurable carryover i.e., the time between the end of block delivery and measuring relative A fibre activity above 90% of baseline was greater than 2 s. A recording from an example trial is shown in Figure 3 to show the stimulation protocol.

### 2.6. Data Analysis

**Figure 4:**
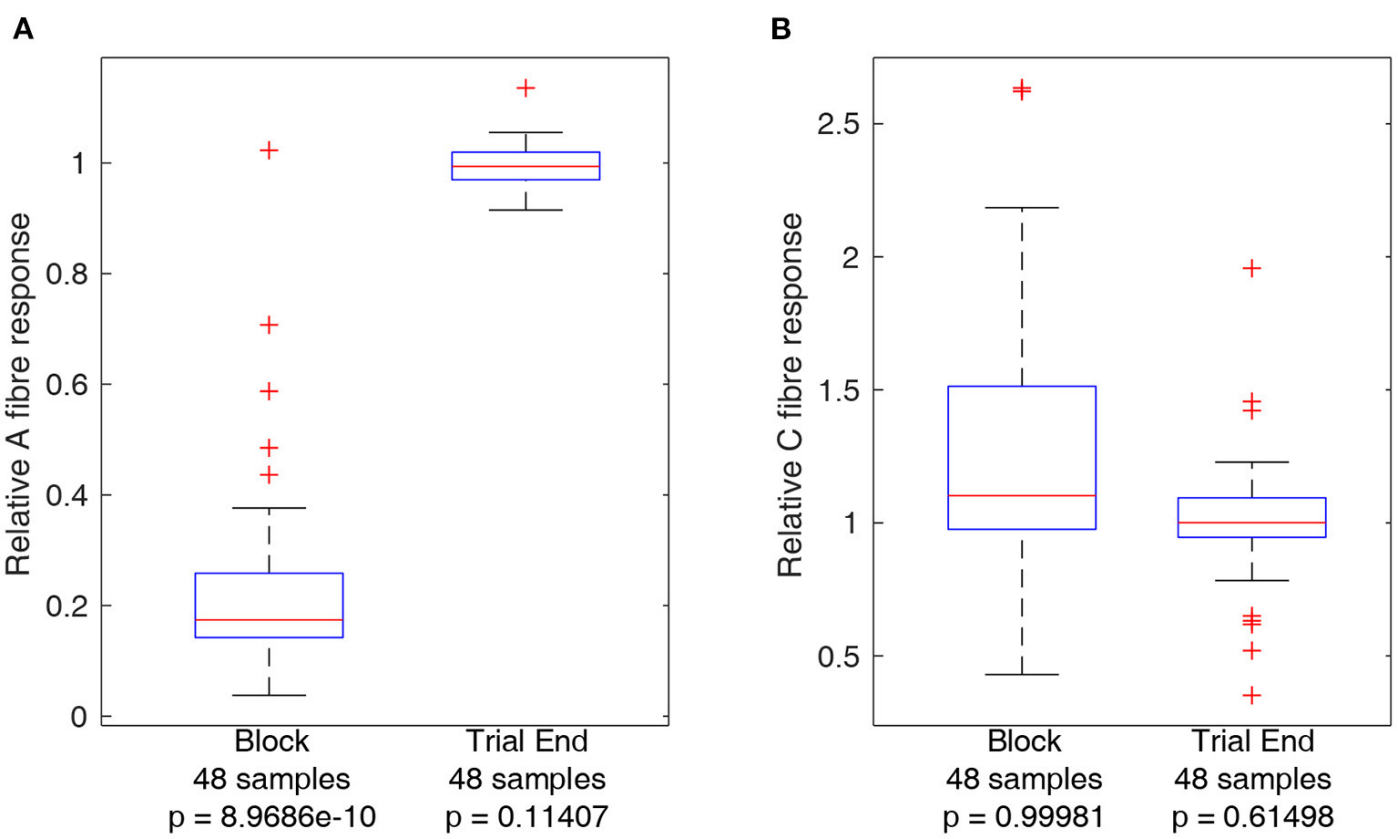
Boxplots comparing A and C fibre activity relative to their respective baselines during block and after recovery form block (trial end). Outliers from each set are identified using red crosses. The horizontal red line for each boxplot represents the median of the set, not including outliers. The number of data points in each set is specified below each boxplot. The *p*-value for a left-tailed sign-rank test relative to 1 is reported below each boxplot. **(A)** Boxplots representing A fibre activity. **(B)** Boxplots representing C fibre activity.

**Figure 5:**
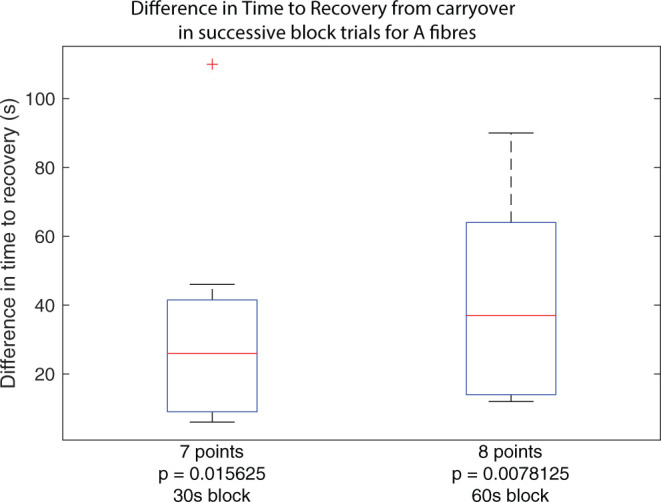
Boxplots of carryover block duration difference between subsequent block trials in the same set across all experiments, for trials using 30 s of block and trials using 60 s of block. Outliers from the set are identified using red crosses. The horizontal red line for the boxplot represents the median of the set, not including outliers. The number of data points in the set is specified below the boxplot. The *p*-value for a right-tailed sign-rank test relative to 0 is reported below each boxplot, testing whether the median of the boxplot set is above 0.

In order to obtain a measure of nerve activity, the trace recorded in Spike 2 (Cambridge Electronic Design) was first duplicated to apply different filtering processes to the A and C fibre CAPs. A fibre CAP signals were filtered within Spike2 using DC removal at 20 ms time constant and smoothing at 100 μs time constant to remove DC offset and high-frequency noise, respectively. Conversely C fibre CAP signals were filtered using DC removal at 20 ms time constant and smoothing at 900 μs time constant respectively. Additionally, stimulation artifacts were digitally blanked in MATLAB (Mathworks) to produce the plots shown in Figure 3. For statistical analysis CAP signals were rectified and integrated in MATLAB to produce a value representing neural activity in response to stimulation. Measures of activity reported in Figures 4, 5 are reported as relative to the baseline activity of 1, comparing activity at start of trials with activity measured during the trial, such as in the block or recovery phases. Signal filtering was carried out in Spike2 (Cambridge Electronic Design) and rectification-integration analysis and plotting were carried out using MATLAB (Mathworks).

## 3. Results

### 3.1. Control Measures: Ensuring Nerve Damage Did Not Occur During Trials

Activity from A and C fibres was measured during block and at the end of the trial (after recovery) to ensure that the nerve had recovered to within 90% of baseline activity. Trials for which this did not occur were not considered in the analyses below and led to termination of the experiment. As the same block settings were used throughout each experiment, it is unlikely application of block itself was the underlying reason for non-recovery of nerve excitability, and the most likely reason is due to progressive loss of function of the nerve outside of the body, an inherent limitation of the *ex-vivo* preparation. Boxplots comparing A and C fibre activity relative to baseline during block and after recovery are plotted in Figure 4. The results show a clear effect of block on A fibre activity while there is no consistent effect on C fibre activity. Measurements showing relative A or C fibre activity above 1 are due to noise, especially for C fibres as using a longer integral to measure nerve activity results in more sensitivity to spurious noise spikes within the recordings. Statistical analysis of A and C fibre activity at the end of each trial shows no significant reduction compared to activity at the start of the trial, indicating delivery of block was safe for the nerve. Reported *p*-values reference left-tailed rank sign tests for each boxplot data set, relative to 1, i.e., testing whether the median of the dataset represented by each boxplot is lower than 1.

### 3.2. Effect of Accumulated Dose on Carryover Duration

While the primary result confirms that carryover applies to A fibres in the rat sciatic nerve, a second observation suggests that subsequent applications of the same dose of HFAC block results in progressively longer carryover, corresponding to an accumulative effect. For trials where carryover was measurable as described previously, considering the same block and stimulation protocol, carryover duration as measured in trial n+1 was subtracted from that measured in trial n, yielding a difference measure for any increase or decrease of carryover duration when repeating trials. Between each trial, A and C fibres were verified to have recovered to at least 90% of baseline activity. The cumulative dose effect holds whether considering applications of block for 30 or 60 s as shown by both boxplots in Figure 5.

The results show that for trials where block carryover is measured to be over 2 s, additional application of block following the same protocol increases the duration of carryover in every case. The increase in seconds spans values from 6 to 110 s. The reported *p*-value under each boxplot corresponds to that of a right-tailed rank-sign test relative to 0, testing whether the true median is above 0, rejecting the null hypothesis.

### 3.3. Carryover Enables Selective Stimulation

A further result from the effect and carryover of HFAC block on A fibres and the lack of effect or carryover on C fibres is selective stimulation of C fibres during the carryover phase of each trial. The presence of A fibre carryover means that there is a window of time starting immediately after the end of block, until the start of significant recovery of A fibre response, during which supramaximal stimulation of the whole nerve only results in measured C fibre activity. This is shown in Figure 3 wherein the C fibre response remains similar during the entire block trial while the A fibre response is significantly suppressed as reported in Figure 4.

## 4. Discussion

In order for stimulation to be made selective using HFAC block, the duration of block carryover for both A and C fibers has to be predicted. There is currently insufficient data to model nerve recovery from carryover in order to make these predictions, although it was shown that the duration is linked to accumulated block duration over several trials in the same experiment. Further work to characterize the effect of block parameters such as amplitude and frequency on carryover remains to be done before a reliable model can be created, however it is also possible that a single variable such as total stimulation charge, dependent on the aforementioned variables can be used to reliably predict block carryover behavior. A further point is that carryover duration is not well-defined with respect to clinical efficacy; in this work it is the time between the end of block and the A fibre response returning to at least 90% of baseline activity as measured from the integrated value of the rectified CAP signal. One goal of improving selectivity is to reduce side-effects of whole-nerve stimulation. This would entail defining a useful carryover window wherein the off-target fibre activity is below a specific threshold, that could be substantially below 90% of baseline. This work provides useful data showing that carryover duration depends not only on the stimulation parameters within a single trial but on the stimulation history of the nerve beyond what can be measured using the CAP peak-to-peak.

To the authors' knowledge there does not currently exist any computational model of nerve that captures HFAC block carryover, or could explain such a process. In previous simulation work (Rapeaux et al., 2015; Perra et al., 2018), nerve fibres with mammalian and amphibian ion channel dynamics both recovered within milliseconds of the block signal being removed. Established models of nerve such as in Frankenhaeuser and Huxley (1964); McNeal (1976), and McIntyre et al. (2002) used in HFAC block simulation studies such as Zhang et al. (2006); Yu et al. (2008); Zhang et al. (2015); Zhao et al. (2015), and Pelot et al. (2017) have never reported observing block carryover *in-silico*. However, not capturing HFAC block carryover *in-silico* is not necessarily due to fundamental limitations of the models themselves, but may be related to assumptions of constant conditions. A typical assumption is that ionic concentrations both inside and outside the nerve remain constant. While this is a reasonable assumption for the simulation of single action potentials over short time scales, this assumption may not be valid when considering the large amount of neural activity occurring as HFAC block is established, and over correspondingly longer time frames as observed experimentally. For example, it has been reported that HFAC block has variable onset duration that can last several seconds in certain cases (Ackermann et al., 2010). Furthermore, With these differences in mind much slower processes can have an impact and must be considered when investigating why carryover occurs.

One compelling hypothesis for an underlying mechanism is accumulation of periaxonal potassium. Initial simulation work by Bellinger et al. (2008) reported how Deep Brain Stimulation (DBS) could result in a depolarization block of axons local to the stimulation electrode due to periaxonal accumulation of potassium. This mechanism is possible due to the presence of potassium channels covered by myelin in the paranodal regions of the axon, next to the nodes of Ranvier at which saltatory conduction occurs. Notably, previous simulation studies have already suggested that these potassium channels play a role in modulating the excitability of nerve fibres, beyond their function as rectifying channels to return the nerve to the resting state after action potential propagation (Baker et al., 1987; Awiszus, 1990). Additional simulation studies such as Brazhe et al. (2011) indicate that high-frequency stimulation in the 100-500 Hz range can result in conduction block due to changes in potassium concentration between the axon and myelin, and a similar process may be occurring with HFAC block, for which the stimulation frequencies are nevertheless significantly higher (Avendano-Coy et al., 2018).

A second point for discussion is a possible limitation of the gold-standard measure used to evaluate nerve excitability. Typically nerve excitability is measured by using peak to peak or integral measures such as the one used in experiments for this work, corresponding to a measure of nerve health or function. Results obtained during experiments show that despite nerve activity having fully recovered to baseline, a repetition of the stimulation protocol used in a previous trial yields different results such as longer carryover. As nerve excitability measures are the same despite different measurements for carryover duration, there has to be some mechanism at work that is not adequately measured by this technique that affects the time needed for nerve fibres to recover from block. Notably, the interpretation by Liu et al. (2013) of a similar observation is that nerve conductibility or excitability cannot be adequately evaluated by measuring the CAP amplitude, and this may also apply to the CAP integral over time. This result is particularly relevant to inform potential clinical applications of this technique for selective stimulation, for example to reduce side-effects of whole-nerve stimulation, which depend on being able to accurately predict the duration of carryover, which determines the amount of time when selective stimulation of C fibres is possible without active block.

Furthermore, while the experiments presented in this work measured carryover for applications of block during 30 and 60 s, due to the structure of the protocol in measuring carryover from repeated trials with the same block parameters, it is not possible to evaluate the effect of HFAC block dosage independently of accumulation. A thorough investigation of the effect of HFAC block dosage on carryover duration should be undertaken, with special care taken to account or control for accumulative carryover effects. Notably, if cumulative effects are non-linear this would either require the use of fresh nerves for every trial comparing each dose level in terms of HFAC duration, amplitude, frequency, or any combination of the above. However, it is possible that cumulative dosage is only observed when the same region of nerve, in the immediate vicinity of the blocking electrode, is blocked multiple times in succession. If this were the case a specific protocol making use of a multi-contact nerve cuff could test multiple doses of HFAC on one nerve in isolation from the effect of accumulated dose. With the present set of measures it was not possible to determine whether accumulated dose effects in carryover only apply locally, or apply to the entire nerve regardless of the location of the blocking electrode, and this question should be answered with further work.

A final point concerns the observation that at times, blocking signal parameters which would reduce A fiber activity to below the noise threshold for supramaximal stimulation parameters, would no longer do so if stimulation amplitude or pulse width was increased beyond the initial values, despite there being no measurable change in A fiber activity. This is in contradiction of results reported previously in Kilgore and Bhadra (2004), where it is specifically asserted that once the block threshold was reached for supramaximal stimuli, any evoked action potentials would not be allowed to conduct. A similar result seems to have been reported in the work of Peh et al. (2018), where block efficacy depended not simply on the block amplitude itself but on the ratio between block amplitude and stimulation amplitude. These observations were inconsistent between experiments, but occurred consistently within the same experiment, and may be related to variability in the nerve-electrode interface as each nerve sample is unique.

In terms of noise, despite the excellent filtering afforded by the oil partition of the setup and the analog filtering and differential recording setup, additional digital filtering of the signal was necessary to improve the quality of measurements such that the block feed-through did not significantly contribute to relative nerve activity. The author is confident the data is representative of actual signal values and not noise, however additional refinements could further improve the quality of quantitative analysis, especially if additional data is collected.

## 5. Conclusion

In this work, we investigated the effect of repeated HFAC block and accumulated charge on carryover block duration in a controlled *ex-vivo* environment. According to measures of CAP integrals, subsequent applications of the same dose of HFAC block led to progressively longer carryover for A fibres. Importantly, A fibre carryover in the absence of C fibre block results in selective activation of A fibres without active HFAC nerve block at supramaximal levels of A fibre recruitment, however clinical applications of this technique would require the duration of carryover to be predictable and the mechanism to be understood. To date this behavior is unexplained, however it is possible a mechanism involving comparatively long-term changes in conditions around the nerve is the root cause. From previous work in the literature and the behaviors observed *in-silico*, a compelling hypothesis is that accumulation of periaxonal or paranodal potassium is responsible for these observations. As the paranode is comparatively isolated from both the axolemma and bulk solution, changes in ionic concentration inside these specific structures, combined with the known role of potassium channels therein in regulating nerve excitability, could explain observed long-term changes in nerve recovery times from HFAC block.

Continued work and investigation of this phenomenon in tightly controlled experimental conditions will enable the isolation of the root cause behind carryover and associated HFAC block dose-based and accumulative effects. One potential avenue for future work is to more closely probe inside the nerve and measure long-term changes in potassium concentration in the paranodal and periaxonal spaces using potassium ion-sensitive microelectrodes as a result of HFAC block, and to compare any observed differences between locations close to the blocking electrode and farther away from it to determine whether mechanisms are local. Ion channel dynamics are well-known in computational models of nerve and could be used to investigate the effect of ion concentration changes on the nerve's ability to initiate action potentials, in combination with experiments. Improved understanding of HFAC block carryover and underlying processes should lead to the development of computational models that reproduce block carryover and predict its duration with subsequent application of HFAC block. This knowledge will enable the use of carryover in clinical scenarios as a useful neuromodulation tool for novel nerve stimulation therapies, or to improve existing therapies by reducing side-effects, for example in vagus nerve stimulation.

## Data Availability Statement

The raw data supporting the conclusions of this article will be made available by the authors, without undue reservation.

## Ethics Statement

The animal study was reviewed and approved by Imperial College Animal Welfare and Ethical Research Board.

## Author Contributions

AR and TC conceived the study, interpreted results, and reviewed and edited the manuscript. AR developed the methodology, carried out experiments, collected and analyzed the data, and wrote the initial draft of the manuscript. Both authors contributed to the article and approved the submitted version.

## Funding

AR was supported through the EPSRC Centre for Doctoral Training (CDT) in High Performance Embedded & Distributed Systems (HiPEDS, EP/L016796/1). This work was also supported by the UK Dementia Research Institute which receives its funding from DRI Ltd, funded by the UK Medical Research Council, Alzheimer's Society and Alzheimer's Research UK.

## Conflict of Interest

The authors declare that the research was conducted in the absence of any commercial or financial relationships that could be construed as a potential conflict of interest.

## Publisher's Note

All claims expressed in this article are solely those of the authors and do not necessarily represent those of their affiliated organizations, or those of the publisher, the editors and the reviewers. Any product that may be evaluated in this article, or claim that may be made by its manufacturer, is not guaranteed or endorsed by the publisher.
